# Clustering Multivariate Time Series Using Hidden Markov Models

**DOI:** 10.3390/ijerph110302741

**Published:** 2014-03-06

**Authors:** Shima Ghassempour, Federico Girosi, Anthony Maeder

**Affiliations:** 1 School of Computing, Engineering and Mathematics, University of Western Sydney, Campbelltown, NSW 2751, Australia; E-Mail: a.maeder@uws.edu.au; 2 Centre for Health Research, University of Western Sydney, Campbelltown, NSW 2751, Australia; E-Mail: f.girosi@uws.edu.au

**Keywords:** health trajectory, HMM, clustering

## Abstract

In this paper we describe an algorithm for clustering multivariate time series with variables taking both categorical and continuous values. Time series of this type are frequent in health care, where they represent the health trajectories of individuals. The problem is challenging because categorical variables make it difficult to define a meaningful distance between trajectories. We propose an approach based on Hidden Markov Models (HMMs), where we first map each trajectory into an HMM, then define a suitable distance between HMMs and finally proceed to cluster the HMMs with a method based on a distance matrix. We test our approach on a simulated, but realistic, data set of 1,255 trajectories of individuals of age 45 and over, on a synthetic validation set with known clustering structure, and on a smaller set of 268 trajectories extracted from the longitudinal Health and Retirement Survey. The proposed method can be implemented quite simply using standard packages in R and Matlab and may be a good candidate for solving the difficult problem of clustering multivariate time series with categorical variables using tools that do not require advanced statistic knowledge, and therefore are accessible to a wide range of researchers.

## 1. Introduction

The interaction of a patient with the health care system takes place at different points in space and time. This implies that in many cases the natural unit of observation for health care research is the entire trajectory of a patient. As linked data and personal health records become more easily available we expect that both researchers and health care stakeholders will have an increasing need for tools that can be used to analyze health trajectories, where by health trajectory we mean a collection of time series describing some aspect of the individual's health or health care utilization.

A very common type of analysis one may need to perform on health trajectories is clustering. For example an insurer may wish to cluster claims trajectories in order to better account for risk categories, a health regulator may need to cluster administrative data trajectories for the purpose of defining appropriate groups for activity based funding, and clinicians may want to group patients with the same condition according to different courses of the disease.

While the analysis of health trajectories is our motivation and our experimental results concern health trajectories, the methods described in this paper do not depend on the fact that the variables we study are related to health, and therefore we will often refer to health trajectories as “multivariate trajectories” or “multivariate time series”.

Clustering multivariate trajectories is a very difficult task, because the notion of cluster is intrinsically linked to the notion of distance, and there is no obvious or standard way of defining a distance between arbitrary multivariate time series. When the time series only contain continuous variables then some well-defined distances can be defined [1,2]. However, when categorical variables are present this is not the case, making it impossible to extend traditional clustering techniques into the time domain. As a result, surveys of the literature [3,4,5] show that most of the clustering methods that make explicit use of a distance are geared toward continuous valued time-series (possibly multi-dimensional) which are much easier to analyze (and visualize) than time series of categorical variables.

Unfortunately, the time series we expect to find in health care related research are likely to contain a mix of categorical and continuous variables. Categorical variables may be used to denote a health condition (such as breast cancer, or diabetes), or a risk factor (such as smoking), the use of medication and the administration of a procedure or of a laboratory test. Continuous variables may arise in conjunctions with claims, costs and results of laboratory tests (such as glucose or cholesterol levels).

As a consequence, in order to make our research relevant for health care applications, in this paper we focus on the issue of clustering trajectories with a method that can handle both continuous and categorical variables at the same time. In addition, we require that the method can be explained in simple terms and implemented without the need of advanced programming or statistical skills, so that it is accessible to a large number of researchers.

Our approach is conceptually simple: since time series of continuous and categorical variables are difficult objects to deal with, we replace each time series with a probabilistic model that is likely to generate it, and then cluster the models, rather than the trajectories, since this task will turn out to be much better defined.

As probabilistic models we use Hidden Markov Models (HMMs) [6], extended to allow for covariates [7,8]. Clustering HMMs is possible because to each HMMs we can attach the likelihood of generating any given trajectory, and the likelihoods can be used to define distances between HMMs. In turns this allows us to use any clustering technique which is based on a distance matrix.

In the terminology of the survey articles [4,5] our approach is referred to as “model based clustering”, and is similar in spirit to the one taken by Ramoni and collaborators [9,10], which is based on Markov Chains rather than HMMs. We will argue in the next section that for applications in health care HMMs with covariates might be a more appropriate starting point than Markov Chains.

For completeness we note that the term “model based clustering” also refers to a different strand of clustering literature, where one assumes that the data were generated probabilistically by a mixture of distributions, each defining a cluster (see [11] and references therein) and a fitting procedure is used to recover the parameters of the distributions. While most of this work has been developed in the static setting, extensions to the trajectory settings have been developed [12,13,14,15,16]. In some of this work [12,13] it is assumed that there is a Markovian dynamic underlying the data and both continuous and categorical variables can be used, like in our approach. However the similarities end there, since we do not make explicit assumptions on the overall probability distribution of the data. Therefore our starting point is really quite different from the one described in [12,13], as it will become apparent in the next section.

## 2. Methods

Our data will be a set of *N* health trajectories *T_i_* corresponding to *N* distinct individuals, where each trajectory is a matrix with *d* columns. Each column is a time series of length *l_i_* that takes values in either categorical or continuous variables. The *d* time series will be in general correlated, and we refer to the variables as the “observables”. The length of trajectories do not have to be the same across individuals, although we assume that we are taking the same *d* measurements for all individuals in the data set.

Standard clustering methods cannot be applied directly to this type of data because the trajectories *T_i_* are not points in a multi-dimensional metric space, and in most cases there is no well-defined notion of distance between trajectories. If we could define a meaningful distance *D*(*T_i_*; *T_j_*) between trajectory *T_i_* and trajectory *T_j_* then clustering would be relatively simple: we could define the distance matrix *D_ij_* ≡ *D*(*T_i_*; *T_j_*) and apply any clustering method that takes as input a distance matrix, such as Partition Around Medoids (PAM) [17], spectral clustering [18] or hierarchical clustering [19]. Therefore a key obstacle to clustering trajectories is the definition of the distance between trajectories.

In the particular case in which the trajectories are continuous valued some natural definitions of distances are available (for example the Frechet distance [1,2]), and some authors have taken advantage of this fact [20,21,22]. However, in most health applications the trajectories will contain categorical variables and there is no obvious metric structure that can be imposed directly on the trajectories themselves.

Therefore we take a different approach: we associate to each trajectory *T_i_* a probabilistic generative model, that is a probability density over the space of trajectories that is likely to generate that trajectory. Then, rather than comparing two trajectories we compare the probability densities associated with them.

This “embedding” approach [23] is attractive because there is a natural way to compare probabilistic models: two models are similar if the probability distributions of the trajectories they generate are similar. Therefore the ill-defined problem of comparing two trajectories can be replaced by the problem of comparing two probability distributions. This problem, while computationally challenging, is well defined and amenable to different solutions, as we will discuss in Section 2.2.

**Figure 1:**
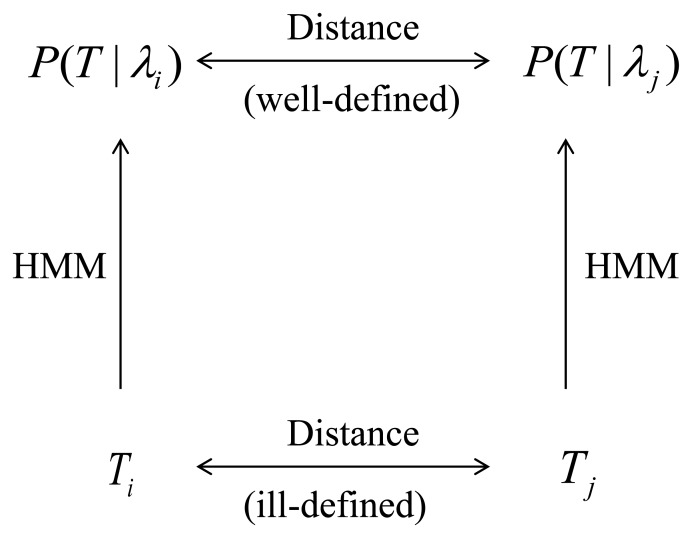
The main premise of this paper is that while the distance between two trajectories is ill-defined, the distance between two probabilistic models that are likely to generate them is well-defined. The class of probabilistic models we consider in this paper is the Hidden Markov Models (HMMs).

The strategy to address the problem of clustering trajectories is therefore as follows:

- We map each trajectory *T_i_* into a probability density over the space of trajectories. In this paper we chose the probability densities to belong to the class of Hidden Markov Models (HMMs) [6,8,24]. We denote the probability density associated to *T_i_* by *P*_λ__*i*_ = *P*(*T*|λ*_i_*), where λ*_i_* is a set of parameters that have been chosen to maximizes the probability of observing the trajectory *T_i_*. We discuss HMMs and methods to estimate their parameters using standard software packages in Section 2.1.
- We define a distance *D*(*P*_λ__*i*_; *P*_λ__*j*_) between probability densities *P*_λ__*i*_ and *P*_λ__*j*_ and define the distance *D*(*T_i_*; *T_j_*) between trajectory *T_i_* and trajectory *T_j_* as *D*(*T_i_*; *T_j_*) ≡ *D*(*P*_λ__*i*_; *P*_λ__*j*_). Figure 1 provides a graphical representation of this process. The distance we use in this paper is the symmetrized Kullback-Leibler (KL) divergence [25]. While the definition of the KL divergence is very standard, its efficient computation can be challenging. In Section 2.2 we address this issue and describe a method that we believe achieves a good compromise between computationally efficiency and accuracy while being easily implemented using standard software packages.
- After having computed the distance matrix *D_ij_* ≡ *D*(*T_i_*;*T_j_*) we perform the clustering of the trajectories by applying a clustering technique that takes as its sole input the distance matrix. In this paper we use the Partition Around Medoids (PAM) [17] method, which is reviewed in Section 2.3.

### 2.1. Hidden Markov Models (HMMs)

Hidden Markov Models (HMMs) are probabilistic models that were introduced in the late 60s [24] and proved to be extremely useful in a variety of disciplines, including speech recognition, weather prediction, financial time series analysis, robotics, detecting protein homologies and computer vision [6,8,26].

HMMs can be thought of as a class of probability densities *P*(*T*|λ) over the space of (possibly multivariate) trajectories *T*, where *P* takes a certain functional form and λ is a set of parameters. HMMs share with the more conventional Markov Chains the assumption that the system under analysis is, at any point in time, in one of a finite number of possible states and that it can transition, at the next point in time, to other states with transition probabilities that satisfy certain Markov properties. However in HMMs the state of the system is “hidden” in the sense that it is not observed. What we observe instead are the values taken by a number of variables, whose probability distributions (usually called *emission probabilities*) are a function of the hidden state.

The simplest example of an HMM is one in which an individual at any point in time is in one of two hidden health states (“sick” and “healthy”) and transitions from one state to another with certain probabilities. The state is not observed, but we observe two variables: body temperature and white blood cell count. The probability distributions of these variables (the *emission probabilities*) depend on the hidden state. For example, the probability of observing high values of body temperature and white blood cell count may be high if the person is in the sick state and low if the person is in the healthy state. While in a traditional Markov model we would model the transition probabilities (and therefore the dynamics) of the observed variables, in this model the dynamics of the observed variables is driven by the dynamics of the hidden states together with the emission probabilities.

The introduction of the hidden states allows HMMs to generate much more complex dynamic patterns than traditional Markov models, while remaining computationally tractable. HMMs are particularly appealing in the health setting because they capture the notion that the health state of an individual is not a well defined quantity, and that the observations available on an individual only capture certain dimensions of health and do not necessarily get to underlying construct of health state. Therefore it seems reasonable to assume that a person's health state remains unobservable, and the only quantities that we can observe are certain “manifestations” of the hidden state.

The notion of hidden state is very much in line with the approach taken, usually in a static setting, by Latent Class Analysis (LCA). Based on the success of LCA in a wide range of applications [27], it seems likely that HMMs will prove to be a valuable tool for the analysis of dynamic health data sets.

In addition, recent advances in HMM theory [8] allow to incorporate easily time-dependent covariates into the model. This means that both the transition and the emission probabilities can depend on a set of covariates, so that they can vary over time and unrealistic stationarity assumption do not need to be made. For example, risk factors such as smoking or obesity status could be used as covariates, since it seems plausible that they influence both the probability of changing the hidden health state and the probability of observing certain health variables given the health state.

Since HMMs are so well-documented in the literature we do not review them in details here. All that matters for the purpose of this paper is the following:

- to each multivariate time series *T_i_* we can associate an HMM model with a given number of hidden states, that is a probability density over the space of trajectories *P*(*T*|λ*_i_*). Here λ*_i_* is a set of parameters that is estimated by maximizing the likelihood *P*(*T_i_*|λ*_i_*) of generating the observed time series *T_i_* and is defined by a triple of parameters: the probability distribution of the initial state, the transition matrix for the hidden states, and the emission probabilities of the observations conditional on the hidden state. We will often refer to the density *P*(*T*|λ*_i_*) simply as “the HMM λ*_i_*”.
- a set of covariates is simply another multivariate time series *z_i_*, of the same length as *T_i_*, that is specific to individual *i*. When covariates are used the transition and emission probabilities are functions of the covariates and therefore are allowed to vary over time.
- the parameters of the HMM λ*_i_* can be estimated easily using statistical software (such as R, Matlab or Mplus). We discuss this in more details in Section 2.1
- Once the parameters of a model λ*_i_* have been estimated, standard statistical software can easily compute the probability *P*(*T*|λ*_i_*) that the model λ*_i_* generates an arbitrary time series *T*.

It is important to notice that current estimation algorithms for HMMs do not estimate the number of hidden states, which must be specified by the user and chosen using some heuristics or additional criteria. We postpone the description of our strategy for estimating the number of hidden states to Section 4.1 since it will be clearer in the context of the experimental results.

#### HMM Estimation

We have used the R package depmixS4 to perform the estimation of the HMMs. Since the problem is unconstrained, parameters are estimated in depmixS4 using the Expectation Maximization (EM) algorithm. We took advantage of the option to introduce covariates in the transition and emission probabilities so that we did not have to make stationarity assumptions. The package allows to fit multivariate time series with both continuous or categorical variables. In our case all variables were categorical and were modeled according to multinomial distributions. Following standard practice we have assumed contemporaneous conditional independence of the multiple time series forming our data ([8], page 123). For categorical data we expect that most HMM users would do the same, since there is no obvious or standard generalization of the multinomial distribution to a multivariate setting, unlike with the normal distribution. The package depmixS4 does not estimate automatically the number of hidden states of the model. The algorithm we have used to this end is explained in Section 4.1 In the next section we will describe how to define and estimate a distance between HMMs models that can be used to define a distance between trajectories.

### 2.2. Definition of Distance between HMMs

The need to compare different HMMs through an appropriate distance measure is not new, and has arisen in a variety of contexts such as speech recognition [28], document and image classification [29], time series prediction [30] and of course in the literature on clustering HMMs [31,32].

In order to define a meaningful distance between two HMMs researchers have used the fact that it is easy to compute, by means of the forward-backward algorithm [28], the likelihood *P(T*|λ) of observing a trajectory *T* given a model λ, and that likelihoods can be used to define distances.

The key observation is that the likelihood *P*(*T*|λ) can be seen as a probability density on the space of trajectories, and a well-defined notion of distance between probability densities already exists, being the Kullback-Leibler (KL) divergence [25]. Therefore the distance between models λ*_i_* and λ*_j_* could be measured as the KL distance between *P*(*T*|λ*_i_*) and *P*(*T* | λ*_j_*), which is:

(1) $$D_{KL}(P(T|\lambda_{i});P(T|\lambda_{j}))\equiv \int \text{dTP}(T|\lambda_{i})\log\frac{P(T|\lambda_{i})}{P(T|\lambda_{j})}$$

The expression above is difficult to compute since it requires to integrate over the space of trajectories, that in most applications will have a high dimensionality Several approaches are available:

- One estimation strategy is a Monte Carlo approach, where one randomly draws a large number *n* of trajectories *T_α_* from the probability density *P*(*T* | λ*_i_*) and then estimates the integral with the finite sum:
  (2) $$D_{KL}(P(T|\lambda_{i});P(T|\lambda_{j}))\approx \frac{1}{n}\sum_{\alpha =1}^{N}\log\frac{P(T_{\alpha }|\lambda_{i})}{P(T_{\alpha }|\lambda_{j})}$$
  This approach suffers from the problem that the number of trajectories one has to drawn in order to achieve a good approximation could be prohibitively large, since the approximation error is of order $n^{-\frac{1}{2}}$. While this does not exclude that in some cases good convergence can be obtained for a reasonably small *n* [33], the applicability domain of this method may be small and highly problem-dependent.
- Alternative methods consist of analytical approaches, such as recursive calculations [34] or fast approximation [35]. However when the HMM have covariates, so that the transition probabilities are time-dependent, is not clear how these methods can be applied, and the programming involved in their implementation may seriously limit the number of potential users.
- On the opposite side of the Monte Carlo approach one could make a strong assumption and estimate the integral in Equation (1) based on one data point only. In order to justify this approach one would have to argue that the probability density *P*(*T* | λ*_i_*) is so highly concentrated around the observed trajectory *T_i_*, that the integral is dominated by the term with *T* = *T_i_*. Under this assumption one can then write
  $$D_{KL}(P(T|\lambda_{i});P(T|\lambda_{j}))\approx \log\frac{P(T_{i}|\lambda_{i})}{P(T_{i}|\lambda_{j})}$$

Since the KL distance is not symmetric one could then “symmetrize” it by writing

(3) $$D_{KL}(P(T|\lambda_{i});P(T|\lambda_{j}))\approx \frac{1}{2}\left(\log\frac{P(T_{i}|\lambda_{i})}{P(T_{i}|\lambda_{j})}+\log\frac{P(T_{j}|\lambda_{j})}{P(T_{j}|\lambda_{i})}\right)$$

Expressions similar to the one of Equation (3) have been used in the literature [32,36,37]. What they all have in common is that they only use the 4 probabilities contained in Equation (3). This approach is simple, but it seems extreme, since it is akin to estimating one very complex integral from one data point, and it is hard to know when the assumption about the concentration of the density may hold.

In order to strike a compromise between the simplicity of Equation (3) (one-point approximation) and Equation (2) (full Monte Carlo approximation) we propose a middle-ground approach. We wish to use many trajectories for the estimation of the integral, like in the Monte Carlo approach, but we want to avoid to have the user to sample the probability densities, which introduces additional complications. Therefore we take advantage of the fact that we already have a sample of trajectories: the entire data set. Our assumption is that the observed data set is sufficiently representative of the universe of possible trajectories. This means that, for any λ ∈ {λ_1_,…,λ*_N_*} we can replace the probability density *P*(*T* | λ), which is defined over the entire space of trajectories, with a discrete probability distribution, defined over the finite set of observed *N* trajectories *T_i_* and represented by a vector *P̃*_λ_ of *N* probabilities that sums up to one:

$$P(T|\lambda )\to {\tilde{P}}_{\lambda }\equiv \frac{1}{Z_{\lambda }}\{P(T_{1}|\lambda ),P(T_{2}|\lambda ),\ldots ,P(T_{N}|\lambda )\}\equiv \left\{\tilde{P}(T_{1}|\lambda ),\tilde{P}(T_{2}|\lambda ),\ldots ,\tilde{P}(T_{N}|\lambda )\right\}$$

where *Z* is a normalizing factor ( $(Z_{\lambda }=\sum_{i=1}^{N}P(T_{i}|\lambda )$) and *P̃*(*T_i_*|λ) ≡ *Z*^−1^*P*(*T_i_*|λ). If we believe that the *N*-dimensional vector *P̃*_λ_ represents sufficiently well the probability density *P*(*T*|λ), then we can define the distance between two HMM models λ*_i_* and λ*_j_* as the KL distance between the two corresponding *discrete* distributions *P̃*_λ__*i*_ and *P̃*_λ__*j*_:

(4) $$D_{KL}(\lambda_{i};\lambda_{j})\equiv D_{KL}({\tilde{P}}_{\lambda_{i}};{\tilde{P}}_{\lambda_{j}})=\sum_{i=1}^{N}\tilde{P}(T_{i}|\lambda_{i})\log\frac{\tilde{P}(T_{i}|\lambda_{i})}{\tilde{P}(T_{i}|\lambda_{j})}$$

As usual with KL distance this expression is not symmetric, and in application it seems best to use its symmetrized version (the average of *D_KL_*(*λ_i_*; *λ_j_*) and *D_KL_*(λ*_j_*; λ*_i_*)). Equation (4) is very simple to compute, since standard HMM packages in R or Matlab easily provide the probabilities involved and the computational cost is quite low. In addition, unlike some other measures proposed in the literature, it takes advantage of all the data, rather than approximating a whole integral with just one point. Therefore we will use Equation (4) as the basis for computing the distance matrix for the trajectories that will be used as input to the clustering method.

### 2.3. Clustering Method

As described in the previous section we define the distance *D_ij_* ≡ *D*(*T_i_*; *T_j_*) between two trajectories *T_i_* and *T_j_* as the symmetrized KL distance between the two corresponding HMMs, λ*_i_* and λ*_j_*:

(5) $$D_{ij}\equiv D(T_{i};T_{j})\equiv \frac{1}{2}(D_{KL}(\lambda_{i};\lambda_{j})+D_{KL}(\lambda_{j};\lambda_{i}))$$

where the KL distance is defined by Equation (4). Once the distance matrix *D_ij_* has been estimated one can apply one of several clustering algorithms (such as Partition Around Medoids [17], spectral clustering [18] and hierarchical clustering [19]) whose sole input is a distance matrix.

For our experiments we have elected to use the Partitioning Around Medoids (PAM) algorithm, a method originally developed by Kaufman and Rousseeuw [38,39,40] that attempts to minimize the dissimilarities between the points that are labeled to be in a cluster and a data point which is designated as the center of that cluster (the “medoid”). Our choice was dictated by the fact that a common problem with clustering algorithms is that they tend to be sensitive to the initial conditions, which makes it often difficult to interpret the results and specify the optimal number of clusters. For PAM we found that the strategy proposed in [41] to remove dependence from the initial conditions was highly effective, and therefore we found it preferable to other methods.

PAM, like other clustering methods, does not determine automatically the optimal number of clusters, which must be chosen by the user. A common approach consists of running a clustering algorithm for different numbers of clusters and computing a “validity index” that assesses the quality of the results for each number of clusters[42]. The number of clusters that corresponds to the best index is then chosen as optimal.

This procedure is particularly useful in cases where there is no prior knowledge of the nature of the clusters [43] and we adopt it here. There is no uniform consensus on which validity index should be used, and therefore we considered three of the most common: the Silhouette index [44], the Davies-Bouldin (DB) index [45] and the Dunn index [46]. The Silhouette index is an average, over all the clusters, of how tightly grouped all the data in each clusters are and it achieves high values for nicely clustered data. The Dunn index and the DB index are somewhat similar, since they both depends on the relative size of the intra-cluster and inter-cluster distance. However, the best Dunn and Silhouette index correspond to the highest value, while the best DB index correspond to the lowest value.

In our experience the Dunn index is the one that has always given the most interpretable and stable results, and therefore this is the one we used in this paper. However, we will also report the Silhouette and DB index, since it turned out that, for our data sets, they actually provide the same answer as the Dunn index and therefore add to the credibility of the results.

## 3. Data

Experiments were conducted using a large, synthetic and complex longitudinal data set, described in Section 3.1, as well as a smaller, real longitudinal survey data set, described in Section 3.2.

### 3.1. Synthetic 45 and up Data

One of the data sets used in our study comes from a simulation based on the 45 and Up Study data [47]. The 45 and Up Study is a survey of the population of the state of New South Wales (NSW), Australia. Prospective participants were randomly sampled from the enrollment database of Medicare Australia, which provides near complete coverage of the population. People 80+ years of age and residents of rural and remote areas were oversampled. A total of 267,153 participants joined the Study by completing a baseline questionnaire (between January 2006 and April 2009) and giving signed consent for follow-up and linkage of their information to routine health databases. About 18% of those invited participated and participants included about 11% of the NSW population aged 45 years and over.

A subset of 60,000 participants was interviewed two to three years after baseline, as part of the Social, Economic and Environmental Factors (SEEF) study. The longitudinal structure of this data set allowed us to estimate transition probabilities between health states over a two years interval.

The health state of an individual was defined by a vector of binary and categorical variables associated to the presence of each of the following chronic conditions and risk factors: heart disease, diabetes, stroke, hypertension, cancer, obesity status and smoking status.

All the transition probabilities of the model were estimated using probit regressions that included as covariates the health state at the previous time, age, gender, income, education, and insurance status. The errors of the terms of the probit regressions are correlated, so that the time series corresponding to different health conditions are correlated and reflect the observed patterns of co-morbidity.

Applying repeatedly the transition probabilities to the original 45 and Up data we obtained a Markov model that we used to generate over 260,000 health trajectories with a time step of two years. The trajectory of an individual stops when the individual dies, where the probability of dying was estimated using data from the NSW Registry of Births, Marriages and Deaths linked to the 45 and Up data.

Out of the 260,000 trajectories, that were originally produced to forecast the health of the NSW population over the next few decades, we extracted a “challenging” subset, which is complex and that exhibits a high degree of variability. We wanted to avoid using trivial trajectories, in which an individual never develops any disease or only develops a condition prior to death, since those are easy to differentiate from the others. Therefore we picked a set of 1,255 trajectories associated with the individuals who developed *all* the three health conditions of heart-disease, stroke and diabetes at some point during their life, and included the obesity and smoking status as time varying covariates. Obesity status for individuals *i* at time *t* was defined by a categorical variable *BMI_it_* taking the values “Underweight”, “Normal”, “Overweight” and “Obese”, obtained by the standard binning of the Body Mass Index (BMI). Smoking status was defined by a categorical variable *Smokecat_it_* taking the values “Not Smoking”, “Smoking” and “Quit Smoking”. We did not include any continuous valued variable because none were available, but it is important to note that HMMs handle continuous and categorical variables equally well, and therefore this is not a concern.

The average age of the cohort is approximately 60, and the length of trajectories is about 18 on average, although it does vary between 4 and 20 time steps, where a time step corresponds to a period of two years of life. The trajectory for individual *i* is therefore represented by five correlated time series (*H_it_*, *D_it_*, *S_it_*, *BMI_it_*, *Smokecat_it_*), which correspond to the variables for heart disease (*H*), diabetes (*D*), stroke (*S*), obesity and smoking status.

We underscore that the probabilistic model underlying these data is not an HMM, it is much more complex than the HMMs used in our experiments and it includes a realistic amount of noise. We resorted to its use only because access to longitudinal health data is limited by ethics and privacy concerns. Despite the fact that this is a simulated data set there is no guarantee a priori that it contains clusters of trajectories, since we have not artificially introduced any. Therefore any clustering structure that we find is a genuine feature of the underlying data.

### 3.2. The Health and Retirement Study

The University of Michigan Health and Retirement Study (HRS) is a nationally representative longitudinal study that has surveyed more than 27,000 elderly and near-elderly Americans since its inception in 1992 [48]. Participants are interviewed every two years, and the study collects data on physical and mental health, insurance coverage, financial status, family support systems, labor market status, and retirement planning. The data set is publicly available and we use the RAND HRS version L, which is an easy to use and cleaned up version of the data [49].

A subset of HRS data with same the characteristics of the synthetic data described in Section 4.1 was chosen. The main difference is the size of sample data set, which is 268, and the length of the trajectories, which is equal to 10. Unlike with the 45 and Up synthetic data the trajectories do not stop when the patient dies, and represent 10 different interviews of live patients.

## 4. Results and Discussion

### 4.1. Results for the 45 and up Study Synthetic Data

For each of the 1,255 trajectories in the 45 and Up synthetic data set we estimate a corresponding HMM using BMI and smoking behavior as time varying covariates. The estimation is performed in R using the package depmixS4 [50], and the number of hidden states was selected to be three. The procedure used to select the optimal number of hidden states is described later, in Section 4.1, since it is best understood after having seen the results.

Once we have estimated the HMMs we used the standard forward-backward algorithm [28] available in the R package depmixS4 to compute the likelihoods *P*(*T_i_*|λ*_i_*), which were then used as input for the algorithms described in Section 2.2 to compute KL distance matrices.

Clustering is then performed using the PAM algorithm. In order to determine the best number of clusters we used the cluster validity indexes discussed in Section 2.3. In Table 1 we show the Dunn, DB, Silhouette indexes as a function of the number of clusters *K* varying from 2 to 10. It is apparent that *K* = 4 is the optimal number of clusters as the Dunn and the Silhouette indexes are maximized, while the DB index reaches its the minimum value.

**Table 1:** The DB, Silhouette and Dunn index for the 45 and Up data, in the case of 3 hidden states. The reason for choosing 3 hidden states is found in Subsection 4.1

**Number of Clusters**	**DB Index**	**Silhouette Index**	**Dunn Index**
2-Cluster	1.6739	0.2074	0.3679
3-Cluster	3.0126	0.1458	0.1815
**4-Cluster**	**1.3980**	**0.2390**	**0.3697**
5-Cluster	2.0816	0.1964	0.2125
6-Cluster	2.9047	0.1291	0.1690
7-Cluster	2.1018	0.1724	0.2176
8-Cluster	2.0894	0.1895	0.1929
9-Cluster	1.9710	0.1651	0.2210
10-Cluster	1.6086	0.2216	0.2601

In order to convince ourselves that four is a reasonable number of clusters for these data we use Multidimensional Scaling (MDS) to visualize the cluster structure. MDS works by finding a set of points in an *d*-dimensional Euclidean space such that the distance matrix of these points is as similar as possible to a given distance matrix D. By choosing *d* equal to two or three one can plot the location of these points and obtain an intuitive representations of how the points that generated the original distance matrix D relate to each other.

**Figure 2:**
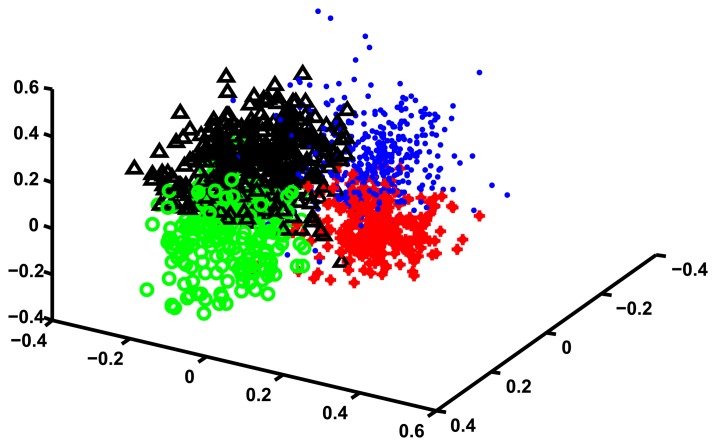
MDS 45 and up.

In our case we choose *d* equal to three, and we find a corresponding set of points in three dimensions. We then plot these points and assign them a unique color depending on which cluster they are in. We manually rotate the plotting view until we find one that best shows the cluster structure. The results of this procedure are shown in Figure 2.

While Figure 2 does suggest that four clusters exists in the data, MDS is only a visual help and does not tell us anything about what is the content of these four clusters, or whether the grouping that we found is sensible. In order to interpret the meaning of the clusters we define, for each trajectory, a set of continuous valued “features” that characterize it, and then compare the means of these features across the four clusters, looking for some meaningful patterns.

An obvious feature that may explain the composition of a cluster is the time *T_c_* spent with a specific health condition *c* (where *c* = *H* for heart disease, *c* = *D* for diabetes and *c* = *S* for stroke). Since the people in the data develop all three conditions the pattern of “features” *T_c_* tells us something about the dynamic of the onset of these three conditions. For example, if a cluster has all high values of *T_H_*, *T_D_* and *T_S_* then people in the cluster developed the three conditions at the same time and early in their trajectory. If instead the values *T_H_*, *T_D_* and *T_S_* are equally spaced then this indicates a steady worsening of health.

Since we are including risk factors in our analysis it also seems important to study how they vary across clusters. The difficulty lies in the fact that both BMI and smoking behavior have significant dynamics that correlate with the onset of disease (for example, some people may lose weight or stop smoking after developing a disease). This implies that reporting the percentage of the *overall* trajectory spent in the different “risk states” (such as being obese or smoking) is not informative. Therefore we constructed an additional set of features that measures the risk factor status before the onset of the first disease (independently of which disease) and after the onset of the third one.

For example, we constructed a feature called “Normal BMI before first disease” that measures the percentage of the portion of trajectory before the first disease that is spent with a normal BMI. If in one cluster this feature is 10% this means that people in that cluster spends only 10% of their time before developing the first disease with normal BMI, and therefore we could label these people as mostly overweight or obese.

Since for each risk factors the corresponding categories are mutually exclusive we report all of them except one. So for smoking we only report the time spent in the “Not-smoking” and “Quit smoking” state, while for BMI we only report the time spent in the “Normal” and “Overweight” state (the “Underweight” category has too few records and is not worth reporting).

**Figure 3:**
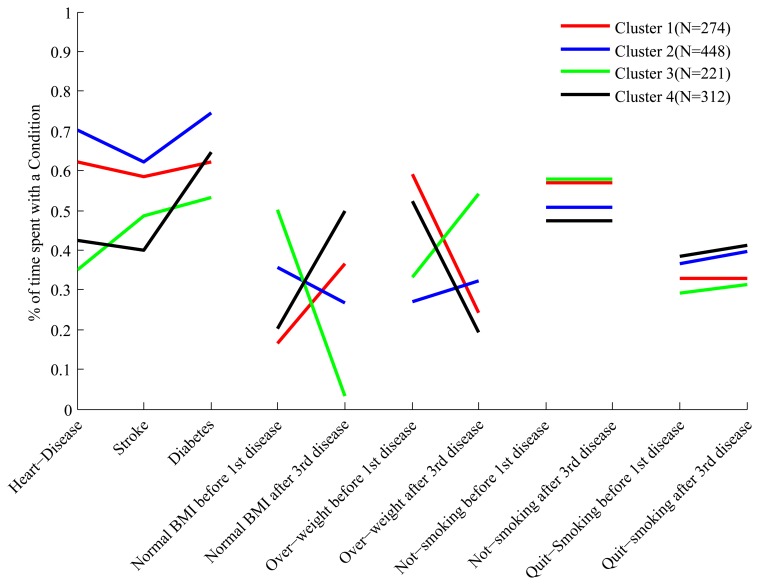
The profile of the four clusters in the feature space for the 45 and Up data.

The full set of features we have constructed for each cluster is shown in Figure 3. Each cluster has a “profile” of features, and the figure clearly shows that while some of the profiles overlap in certain features, they are all distinct.

In order to make it easier to interpret the composition of the clusters based on their feature profile we describe some clusters in details. Looking at the first three features of cluster 4 we see that people in this cluster spend about 60% of their trajectories with diabetes, and 40%–45% of their trajectories with heart disease and stroke. Therefore these people develop diabetes relatively early, and after a period of time develop both heart disease and stroke. This is quite different from what we observe, for example, in cluster 1, where people spend about 60% of their trajectories with all three conditions.

Still in cluster 4 we notice, by looking at the feature labeled “Normal BMI before first disease”, that these people, before developing their first condition (which is diabetes), spend only 20% of their trajectories in the state of “Normal BMI”, and therefore are mostly overweight and obese. However, looking at the feature labeled “Normal BMI after third disease” we notice that after developing the third disease these people spend at least 50% of their trajectories with normal BMI, and therefore a significant proportion experiences weight loss after the heart and stroke events. This is the opposite of what we observe for cluster 3, whose constituents are of relatively normal BMI before the first disease (diabetes) but experience weight gain after developing heart disease.

We have summarized the features of the different clusters in Table 2, for the purpose of showing that an interpretation of the composition of the clusters is indeed possible. Before moving to the analysis of the real data, however, we describe how we arrived at the conclusion that the best HMM for our problem has three hidden states.

**Table 2:** Interpretation of the four clusters for the 45 and Up synthetic data. Note that the expressions such as “mostly”, “significant” or “some” do not refer to the size of the effect on an individuals, but rather to the size of the population that experiences the effect. Therefore “Some weight gain” means that some of the the people in the cluster experiences weight gain. Interpretation for not-smoking behavior omitted because of lack of change.

	**Disease onset**	**Normal BMI**	**Overweight**	**Quit smoking**
**Cluster 1**	Heart disease, stroke and diabetes almost simultaneously	Mostly overweight/obese before 1st disease	Some weight loss after 3rd disease	No change in smoking behaviour after 3rd disease
**Cluster 2**	Diabetes, heart disease and then stroke	Significantly overweight/obese before 1st disease	Some weight gain after 3rd disease	Mild increase in quitting smoking after 3rd disease
**Cluster 3**	Diabetes, stroke and then heart disease	Half time normal BMI before 1st disease	Significant weight gain after 3rd disease	Mild increase in quitting after 3rd disease
**Cluster 4**	Diabetes and then heart disease and stroke	Mostly overweight/obese before 1st disease	Significant weight loss after 3rd disease	Mild increase in quitting after 3rd disease

#### Choosing the Number of Hidden States

The algorithms that estimate the parameters of the HMMs do not estimate the optimal number of hidden states. This parameter needs to be chosen by the users according to some criterion or some additional prior knowledge. We use the fact that we can represent the clusters as “feature profiles”, as shown in Figure 3, to define a criterion of optimality. Our criterion is that the feature profiles should be as uncorrelated as possible, so that it is easy to see the difference across clusters in the feature space. Therefore we proceed as follows. For a given tentative number of hidden states we perform the clustering, select the optimal number *K* of clusters according to the Dunn index described in Section 2.3, and then compute the average of the correlation coefficients between the $\frac{K(K-1)}{2}$ possible pairs of the *K* feature profiles. This procedure is repeated for several possible values of hidden states, and the number of hidden states corresponding to the lowest average correlation is selected as optimal. The result of this procedure is shown in Table 3, where the number of hidden states varies between the minimum number, two, and four. The table clearly shows that the optimal number of hidden states, according to this criterion, is 3.

**Table 3:** The optimal number of hidden states for the 45 and Up synthetic data is 3, since it corresponds to the lowest average correlation across the feature profiles.

**Number of Hidden states**	**Number of clusters**	**Correlation**
2 Hidden states	*K* = 2	0.74
**3 Hidden states**	***K* = 4**	**0.41**
4 Hidden states	*K* = 3	0.47

The reason for which we do not consider more than 4 hidden states is that for a larger number of states the number of parameters of the HMMs would be too high, relative to the length of the trajectories, and over fitting would most certainly occur. The general manifestation of over-fitting would be instability [51]: two trajectories that are almost identical could end up being mapped in two densities that are far apart, with disastrous consequences for the clustering algorithm. Restricting the number of hidden states under consideration is a crude method for avoiding over-fitting. More sophisticated methods include cross-validation, regularization and Bayesian integration, as described in [52].

Cross-validation involves using only a portion of a trajectory to estimate the parameters of the HMM, using the remaining portion to check whether the HMM predicts the trajectory correctly. This is easy to implement, although it is probably not meaningful for short time series and not practical for large data sets. Regularization involves setting restrictions on the set of parameters that need to be estimated. The R package depmixS4 allows to estimate the HMMs under inequality constraints, and therefore this option may be attractive for a reasonably wide range of users. The Bayesian integration method is much more involved than the other two, and it would be probably appealing to a restricted set of users.

### 4.2. Results for a Synthetic, Validation Data Set

A standard way of testing whether a novel clustering method performs as expected is to test it on a validation data set, for which one already knows the structure of the data, so that an appropriate comparison can be made. In our case this means to apply the algorithm to a set of trajectories that have been generated by HMMs with a known number of hidden states and that are expected to belong to a given number of clusters. The validation consists in checking that we recover the correct number of hidden states and cluster structure.

This type of experiments is particularly informative when there is an agreed notion of how difficult the validation task is, and often there are benchmark data sets that can be used to this end. In our case no such data set exists, and there is no obvious notion of “difficulty” of a data set. Therefore any test we may run is more of a sanity check than a validation test, in the sense that if the algorithm failed we would have to investigate why, but if it worked successfully we could not draw any conclusions since it is possible that the problem is simply not challenging enough.

Nevertheless it seems worth to run such type of test, especially on a data set that does not appear trivial. Therefore, rather than constructing an arbitrary, new synthetic data set we took advantage of the results of the previous section to generate a non trivial data set. In the previous section we showed that our method found 4 clusters in a set of 1,255 trajectories, and estimated that the optimal number of hidden states that describe the data is 3. In order to produce our artificial data set we took the 4 trajectories corresponding to the centers of the 4 clusters. To each of them it corresponds an HMM with three hidden states, and therefore a probability density *P*(*T*|λ*_i_*), *i* = 1,…,4. We sampled each density to produce as many sampled trajectories as in the original clusters (as shown in Figure 3).

The resulting set of 1,255 trajectories is all generated by HMMs with 3 hidden states, and if we applied our algorithm we should be able to recover the fact that there are 4 clusters in the data and that the optimal number of hidden states is 3. This is indeed the case, as shown in Table 4.

**Table 4:** The optimal number of hidden states for the validation data set is 3, since it corresponds to the lowest average correlation across the feature profiles. This is indeed the number of hidden states used to generate the data.

**Number of Hidden states**	**Number of clusters**	**Correlation**
2 Hidden states	*K* = 5	0.12
**3 Hidden states**	***K* = 4**	**0.07**
4 Hidden states	*K* = 3	0.17

In addition, trajectories sampled from the same HMM λ*_i_* should be classified as belonging to the same cluster, at least approximately. We do not expect perfect agreement, since the cluster centers might be close to each other and the clusters may easily overlap, leading to some misclassification. In order to quantify the ability of the algorithm to group trajectories sampled from the same density we constructed a confusion matrix [53], that we show in Table 5. On the rows of the confusion matrix we have the “actual class”, that is the label of the HMMs from which a trajectory is sampled. On the columns we have the “predicted class”, that is the label provided by our clustering algorithm. The labeling of the four cluster found by our algorithm is clearly arbitrary, and we simply choose a labeling that makes it easy to look at the results. The table shows, for example, that out of the 274 trajectories sampled from HMM 1, 199 were in the same cluster, that we conveniently labeled “Cluster 1”, while the others were assigned mostly to clusters that we labeled “Cluster 2” and “Cluster 4”. Similarly, all the 312 trajectories sampled from HMM 4 were labeled “Cluster 4”. Notice that with our choice of labeling for the clusters found by the algorithm the confusion matrix is nearly diagonal. This highlights the fact that the algorithm reconstructs the original structure quite well, with a relatively small number of misclassifications.

**Table 5:** Confusion matrix between true cluster and predicted cluster results.

		**Predicted class**			

		**Cluster 1**	**Cluster 2**	**Cluster 3**	**Cluster 4**
**Actual class**	**Cluster 1**	199	49	1	25
	**Cluster 2**	5	441	2	0
	**Cluster 3**	0	0	221	0
	**Cluster 4**	0	0	0	312

### 4.3. Results for the Health and Retirement Study

**Figure 4:**
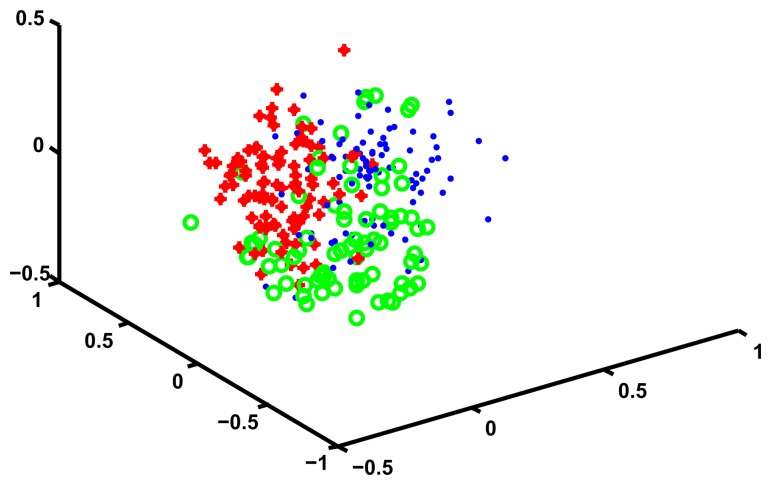
MDS HRS data.

The analysis for the HRS data follows exactly the same lines of the analysis of the 45 and Up data. The optimal number of hidden states turned out to be 3, as shown in Table 6. In Table 7 we show the Silhouette, the Dunn and the DB index for varying numbers of clusters. The table clearly shows that *K* = 3 is the optimal number of cluster for the HRS data, since the Dunn and the Silhouette indexes are maximized, while the DB index is minimized. We use MDS to visualize the three clusters and show the results in Figure 4.

**Table 6:** The optimal number of hidden states for the 45 and Up data is 3, since it corresponds to the lowest average correlation across the feature profiles.

**Number of Hidden states**	**Number of clusters**	**Correlation**
2 Hidden states	*K* = 2	0.59
**3 Hidden states**	***K* = 3**	**0.53**
4 Hidden states	*K* = 4	0.62

**Table 7:** The DB, Silhouette and Dunn index for the HRS data, using 3 hidden states.

**Number of Cluster**	**DB Index**	**Silhouette Index**	**Dunn Index**
2-Cluster	2.0529	0.4636	0.3893
**3-Cluster**	**1.5465**	**0.4645**	**0.5310**
4-Cluster	2.1257	0.4412	0.2820
5-Cluster	1.8561	0.3974	0.2614
6-Cluster	2.6350	0.4321	0.1841
7-Cluster	1.8339	0.4304	0.3497
8-Cluster	2.6883	0.4506	0.2170
9-Cluster	2.5222	0.4536	0.1544
10-Cluster	2.2139	0.4226	0.2243

**Figure 5:**
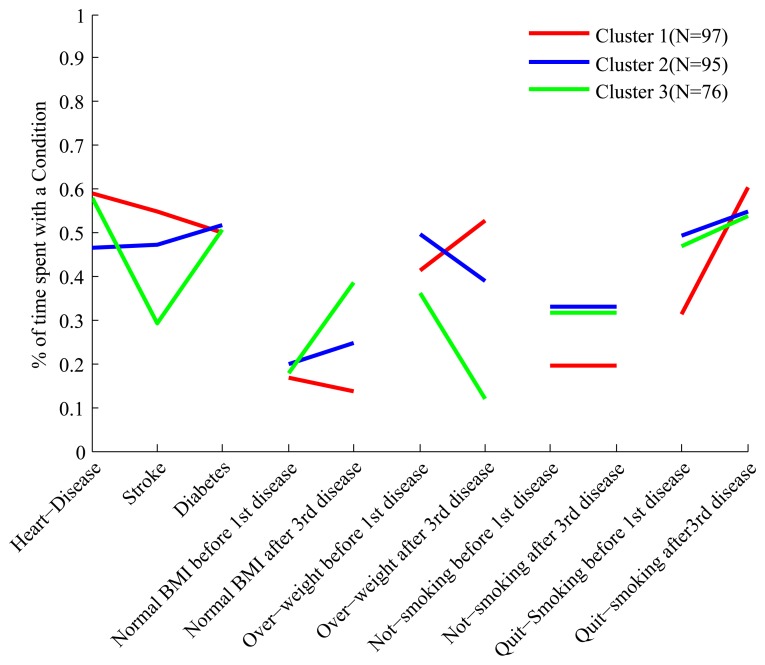
The profile of the three clusters in the feature space for the HRS data.

In order to interpret the clusters we use the same methodology we used for the 45 and Up data, and in Figure 5 we show the profiles of the clusters in the feature space. The three clusters are well separated and are easy to interpret. A description of the clusters is presented in Table 8, but in order to facilitate the interpretation we describe cluster 3 in more detail.

**Table 8:** Interpretation of the three clusters in the HRS data. Note that the expressions such as “large” or “mostly” do not refer to the size of the effect on an individuals, but rather to the size of the population that experiences the effect. Therefore “Large weight loss” means that a large portion of the people in the cluster experiences weight loss. Interpretation of not-smoking omitted because of lack of change.

	**Disease onset**	**Normal BMI**	**Overweight**	**Quit smoking**
**Cluster 1**	Heart disease, then stroke and then diabetes	Mostly overweight or obese before 1st disease	Weight gain after 3rd disease	Large increase in smoke quitting after 3rd disease
**Cluster 2**	Diabetes and then heart disease and stroke almost at the same time	Mostly overweight or obese before 1st disease	Some weight loss after 3rd disease	Increa1se in smoke quitting after 3rd disease
**Cluster 3**	Heart disease, then diabetes and much later stroke	Mostly overweight or obese before 1st disease	Large weight loss after 3rd disease	Increase in smoke quitting after 3rd disease

People in cluster 3 spends 60% and 50% of their trajectories with heart disease and diabetes respectively, so that they develop first heart disease and diabetes soon after. Unlike people in other clusters they develop stroke much later, and spend only 30% of their trajectory with that condition. A sizable proportion of people in this cluster experiences weight loss and reverts to normal BMI after developing stroke, unlike people in cluster 1 who may actually gain weight. Compared to people in cluster 1 they are less likely to be non-smokers before the first disease, and a moderate proportion of them quits smoking after developing stroke.

We are aware that these interpretations are not complete and many other factors (starting from age) need to be looked at, but this is not in the scope of the paper. The purpose of these interpretations is simply to show that these clusters are well separated and that correspond to some meaningful groups of individuals.

## 5. Conclusions

While there is significant literature on the problem of clustering time series of continuous variables [3], much less is known when the time series consist of categorical variables. We anticipate that, as more and more longitudinal data sets become available, many of the time series to be clustered will consists of a mix of continuous and categorical variables. This seems particularly true in the domain of health, where categorical variables are ubiquitous and may be associated with health and functional status, risk factors and diagnostic/procedure codes. Hence, in this paper we have described a clustering method that is indifferent to the distinction of continuous *vs*. categorical.

Our goal was to devise a method that is sound, easy to explain and that can be implemented easily using statistical software such as R or Matlab, taking advantage of already existing packages. While it is true that research in health care is becoming more and more interdisciplinary it seems that a method for clustering health trajectories that requires users to write their own Monte Carlo Markov Chains or dynamic programming routines would be of little use to the community of practitioners.

We believe the methodology is sound because it is based on the simple idea of representing a complex, unstructured object (a multivariate time series) with a well defined dynamic model: embedding data in generative models is a common theme in the machine learning literature, and it is a well-tested strategy [23].

Our approach to compute distances between HMMs is based on the notion of KL distance, and it is standard in the literature. The innovation we bring over methods such as those described in [32,36,37] is that we use much more information, without requiring the user to perform more complex operations: all that is required is to compute the likelihood that an HMM generates a given trajectory, which is a basic operation of any HMM software package.

The advantage of our procedure over more sophisticated methods, such as those based on recursive calculations [34] or fast approximation [35] is that it applies easily to the case of HMMs with covariates and it is also much easier to implement. The use of covariates is bound to be common in health care applications, since we would not always expect the transition probabilities underlying the data to remain constant over time (for example, they may be age dependent).

We have performed three tests on our method. For the first test we used a synthetic data set based on the 45 and Up and SEEF surveys, which consisted of 1,255 trajectories of people over age 45, with an average trajectory length equal to 18. The simulation that generated the data was complex and it was not built to contain any clusters. We applied our method to a non-trivial set of trajectories, where all subjects develop three chronic conditions and where the transition probabilities across hidden states depend on two time varying covariates (obesity and smoking). The method proposed allowed to identify 4 clear clusters of records that differed in the dynamics of the chronic disease as well of the risk factors.

For the second test we used the results of the first test to built a simulated set of four clusters of trajectories generated by HMMs with 3 hidden states. We tested our method by checking that we recovered the correct clustering structure and number of hidden states.

For the third test we used a subset of the Health and Retirement Survey, a longitudinal study of people over age 50. This set consisted of 268 trajectories of length 10, with the same variables as the simulated data. Our method was able to identify 3 clear clusters that could be easily interpreted.

The main point of this paper was not to solve a specific problem, but to show a well-grounded method for clustering health trajectories that can be implemented quite simply with statistical software such as R or Matlab. All that is required to implement this method is a package that can estimate an HMM (possibly with covariates) given a multivariate continuous or categorical time series and that can compute the likelihood of a trajectory given an HMM. Once these two components are in place then clustering can be performed with any method that takes as input a distance matrix. We found PAM [17], with the initialization procedure described in [41], particularly effective, but other methods, such as spectral clustering [18] and hierarchical clustering could be used as well.

The main limitation of this method is that HMMs are not particularly meaningful for very short trajectories, since even for a small number of hidden states one could easily end up having to estimate more parameters than the number of observations. Therefore we do not expect it to work well if one only has few time periods. In addition, while the method worked well with few thousand observations it would run into difficulties with a very large number of observations, say one million, since it would require to estimate one million HMMs and then run PAM on a distance matrix with one million rows. Clearly for large data sets some partitioning or a hierarchical approach, such as the one proposed in [54], will have to be developed.
